# Entanglement Swapping Enables the Practical Security of Quantum Cryptography

**DOI:** 10.3390/e28050518

**Published:** 2026-05-04

**Authors:** Yang-Fan Jiang, Liang Huang, Yu-Zhe Zhang, Likang Zhang, Qi-Chao Sun, Zheng-Ping Li, Hao Li, Weijun Zhang, Lixing You, Feihu Xu, Qiang Zhang, Jian-Wei Pan

**Affiliations:** 1 Hefei National Research Center for Physical Sciences at the Microscale and School of Physical Sciences, University of Science and Technology of China, Hefei 230026, China; 2 Jinan Institute of Quantum Technology, Jinan 250101, China; 3 Hefei National Laboratory, University of Science and Technology of China, Hefei 230088, China; 4 China Telecom Quantum Information Technology Group Co., Ltd., Hefei 230088, China; 5 Shanghai Research Center for Quantum Science and CAS Center for Excellence in Quantum Information and Quantum Physics, University of Science and Technology of China, Shanghai 201315, China; 6 Shanghai Key Laboratory of Superconductor Integrated Circuit Technology, Shanghai Institute of Microsystem and Information Technology, Chinese Academy of Sciences, Shanghai 200050, China

**Keywords:** quantum cryptography, entanglement swapping, entangled photon pairs

## Abstract

Entanglement is one of the most striking phenomena in quantum physics, playing important roles in fundamental physics and quantum information science. It enables a secure means of communication—quantum cryptography—and builds up the foundation of its unconditional security. Entanglement-based quantum cryptography has received great attention from the early demonstrations to the recent remarkable achievements. In a practical scenario, although entanglement-based quantum cryptography can provide inherent source-independent security, its detection side has been shown to be vulnerable to external probing attacks. Here we show that entanglement swapping can effectively solve this critical issue, enabling a side-channel-free quantum cryptography.Entanglement swapping allows each user’s quantum state preparation and detection in a completely private station, which is immune to *any* external probing side channels. We demonstrate the entanglement-swapping quantum cryptography scheme in the field based on two independent entanglement photon sources. Based on the remote entangled photon pairs, we implement the Ekert-1991 protocol under a channel attenuation equivalent to 100 km of standard optical fiber, achieving a Bell violation value of S=2.659±0.092 and a secret key rate of 0.0163 bit/s. While recent device-independent QKD demonstrations have reached 100 km using atoms or ions, our photonic ES-QKD offers a complementary, all-optical pathway that is directly compatible with existing fiber networks and quantum repeaters.

## 1. Introduction

Quantum key distribution (QKD) [1,2,3,4] promises a secure means of unconditionally secure communication [5,6]. In theory, the security has been proven based on the fundamental laws of quantum physics [7,8]. In practice, however, the devices implementing QKD were shown to have imperfections [9,10,11,12,13], which might be exploited by attackers. For this reason, an arms race has been going on during the past two decades among quantum code-makers and quantum code-breakers [5].

**Figure 1:**
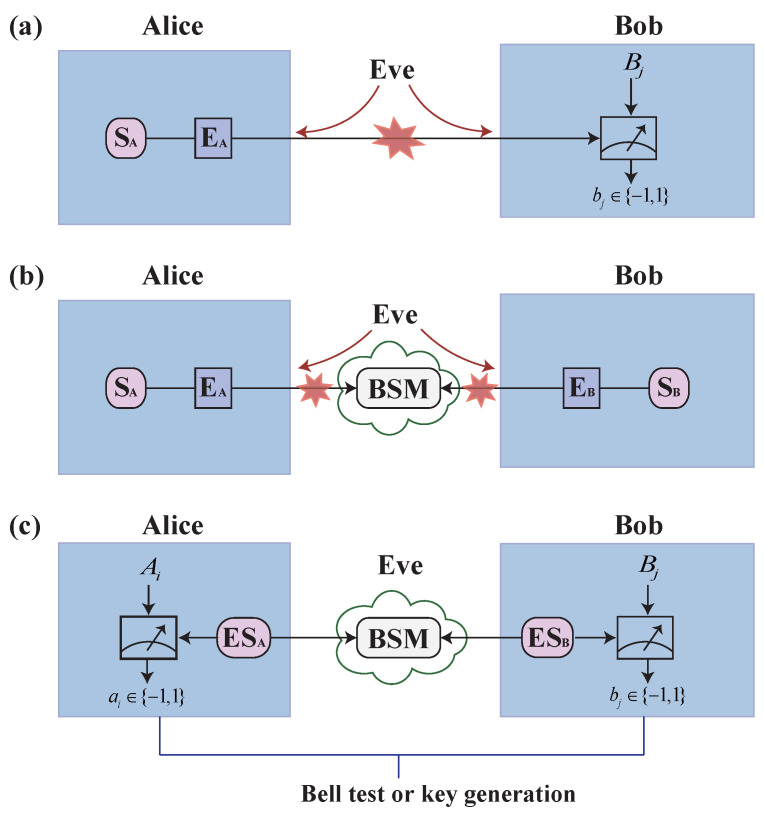
Schematic diagrams of different quantum key distribution (QKD) protocols. (**a**) Standard BB84 QKD system, which is vulnerable to Trojan horse attacks against the source and detector-control attacks against the detection. Here, $S_{A}$ ($S_{B}$) and $E_{A}$ ($E_{B}$) represent the source and encoder at Alice’s (Bob’s) side, respectively, and the red stars indicate the vulnerabilities to side-channel attacks. (**b**) Measurement-device-independent QKD (MDI-QKD) protocol, which resolves all side-channel vulnerabilities in the detection part. (**c**) The proposed entanglement-swapping QKD (ES-QKD) scheme. Alice and Bob generate entanglement sources ($ES_{A}$ and $ES_{B}$) and perform random and independent measurements on half of the photon pairs within their secure labs. $A_{i}$ ($B_{j}$) denotes the measurement basis, and $a_{i},b_{j}\in \left\{-1,1\right\}$ are the corresponding measurement outcomes. An untrusted node (Eve) performs Bell state measurement (BSM) on the photons sent from Alice and Bob. Following entanglement swapping, Alice and Bob implement the Bell test and generate secure keys. This scheme ensures that each user’s state preparation and detection occur in a completely private space, providing physical immunity against external probing side channels.

For a practical QKD system, broadly speaking, any port can potentially allow side channels to probe the source settings and the detection settings. For instance, a standard BB84 QKD system (Figure 1a) suffers from both the Trojan horse attacks (THA) against the source [14,15,16] and the detector-control attacks against the detection [11,12,13]. Remarkably, the proposal of measurement-device-independent QKD (MDI-QKD) [17] resolves all the side channels in the detection part (Figure 1b), thus greatly enhancing the security of practical QKD. An efficient MDI-QKD method, namely twin-field QKD [18], can improve the transmission distance. Nonetheless, MDI-QKD is subject to subtle side channels for the source such as the THA [14,15,16] and laser-seeding attacks [19,20].

A secure QKD system must effectively isolate any physical side-channel probes from *both* the source and the detection. While fully device-independent QKD (DI-QKD) [21,22,23] offers the ultimate theoretical solution, its stringent requirements on overall efficiency have long restricted its transmission distance. Very recently, milestone experiments have successfully extended DI-QKD to the 100-km scale utilizing single-atom nodes [24] and trapped-ion quantum memories [25]. While these matter-qubit architectures provide the long-lived entanglement crucial for quantum repeaters, they inherently require sophisticated trapping and cooling setups. A highly scalable, purely photonic approach relying entirely on standard telecommunication infrastructure remains an essential parallel objective.

Remarkably, the side-channel-free QKD based on entanglement swapping [26,27,28] (Figure 1c) enables each user’s quantum state preparation and detection in a completely private space, where any probe from outside is perfectly isolated from the state-generation [1]. Similar to the idea of teleportation filter [7], each user’s private space receives only classical signals without any quantum signals from outside. This allows physically protecting the user’s devices from any external probing. We call this scheme entanglement-swapping QKD (ES-QKD).

Here we perform an experimental demonstration of the ES-QKD scheme in the field. Different from previous entanglement-swapping experiments that were realized with the same pump locally [28,29,30,31,32], we develop two truly independent entanglement sources in the real world via active stabilization strategies. Based on the remote entangled photon pairs, we implement the Ekert-91 protocol [2] over a 50 km coiled fiber and under a simulated attenuation equivalent to 100 km, achieving secret key rates of 0.121 and 0.0163 bit/s, respectively. Furthermore, we comprehensively evaluate the finite-key security and meticulously characterize the optical isolation of the system. Besides the advantage of enhanced security, our QKD scheme is compatible with the structure of device-independent QKD [22,23] and quantum repeaters [33,34].

## 2. Protocol

Figure 1c shows the scheme of the ES-QKD protocol. Alice and Bob prepare entangled photon pairs in their sites and share them with an untrusted node (in the worst cases, the node is controlled by Eve). Then the remote photons held by Alice and Bob are entangled conditioned on the outcomes of the Bell state measurement (BSM) on the photons paired with them performed by Eve, i.e., via entanglement swapping [26,27,28]. As discussed in detail in Ref. [1], after the entanglement swapping, Eve has no access to the entangled photon pairs shared between Alice and Bob anymore. Moreover, stemming from the nonlocal property of entangled states, Eve’s knowledge about swapped entanglement can be bounded by the violation of Bell’s inequalities [2]. To obtain secure keys, Alice and Bob randomly and independently choose their measurement basis $A_{i}\in \left\{\sigma_{z},\frac{1}{\sqrt{2}}\left(\sigma_{z}-\sigma_{x}\right),\frac{1}{\sqrt{2}}\left(\sigma_{z}+\sigma_{x}\right)\right\}$ and $B_{j}\in \left\{\sigma_{z},\sigma_{x}\right\}$, respectively, where $\sigma_{z}$ and $\sigma_{x}$ are two Pauli matrices. The coincident detection results with basis $\frac{1}{\sqrt{2}}\left(\sigma_{z}-\sigma_{x}\right)$ or $\frac{1}{\sqrt{2}}\left(\sigma_{z}+\sigma_{x}\right)$ and $\sigma_{z}$ or $\sigma_{x}$ are used to estimate the violated CHSH-type Bell’s inequality with

(1) $$S=\;|\langle A_{1}B_{1}\rangle +\langle A_{1}B_{2}\rangle +\langle A_{2}B_{1}\rangle -\langle A_{2}B_{2}\rangle |,$$

where $\langle A_{i}B_{j}\rangle =P\left(a_{i}=b_{j}|A_{i}B_{j}\right)-P\left(a_{i}\neq b_{j}|A_{i}B_{j}\right)$, $a_{i}$ and $b_{j}$ are binary outputs of measurement $A_{i}$ and $B_{j}$. If Alice and Bob do not achieve the expected violation, they abort the protocol. If they both measure in the basis $\sigma_{z}$, the measurement outcomes are strongly correlated and will serve as a raw key. In the asymptotic limit, the secret key rate against collective attacks is given by [23]

(2) $$\begin{array}{c} r\geq 1-h\left(\frac{1+\sqrt{{\left(S/2\right)}^{2}-1}}{2}\right)-h\left(Q\right) \end{array}$$

where *h* is the binary entropy and *Q* is the quantum bit error rate (QBER).

While Equation (2) provides the theoretical limit in the asymptotic regime, practical QKD systems operate for a limited duration and yield a finite number of data blocks. To guarantee practical security, it is essential to consider the finite-size regime, shown in Appendix A, which provides a rigorous and realistic secret key rate by accounting for the statistical confidence intervals of the Bell test and the intrinsic error probabilities of classical post-processing.

## 3. Experiment

**Figure 2:**
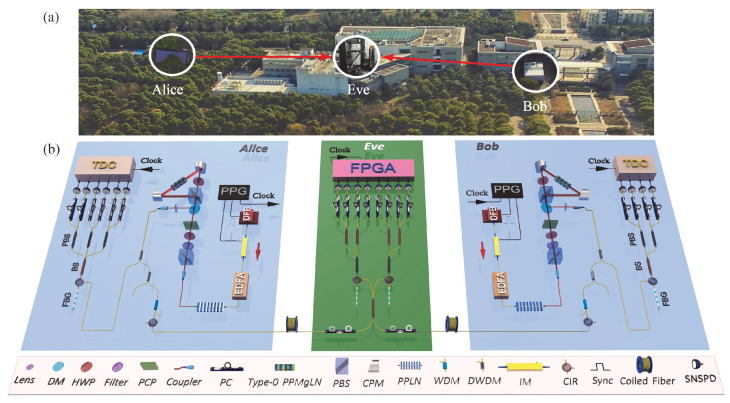
(**a**) Birds-eye view of the experiment. (**b**) Experimental setup. Alice’s (or Bob’s) setup: She generates laser pulses using a 1558 nm gain-switched distributed feedback laser (DFB) and an intensity modulator (IM), which are driven by a pulse pattern generator (PPG). The pulses are amplified by an erbium-doped fiber amplifier (EDFA) and up-converted to 779 nm in a periodically poled lithium niobate (PPLN) crystal. The produced 779 nm pulses are focused into the Type-0 PPMgLN in a Sagnac loop to generate polarization-entangled photon pairs. The signal and idler photons are singled out by an inline dense wavelength division multiplexing filter (DWDM). The signal photon is further filtered by the wavelength division multiplexing filter (WDM) and through a circulator (CIR), and sent to Eve. The idler photon is measured locally. Alice and Bob randomly employ three and two measurement bases on photons by the beam splitters (BSs), respectively. And the results are recorded by the time-to-digital converters (TDCs). Eve’s setup: Eve performs the BSM with the combination of 50:50 BSs, polarization beam splitters (PBSs) and eight superconducting nanowire single-photon detectors (SNSPDs). The BSM results are analyzed in real-time and recorded by a field-programmable gate array (FPGA). All devices are synchronized by the clock signals from Alice’s PPG. Abbreviations of other components: HWP, half-wave plate; PC, polarization controller; PCP, phase compensator plate; OPM, off-axis parabolic mirror; DM, dichroic mirror.

A schematic of our experimental apparatus is shown in Figure 2. We construct a three-node ES-QKD system in the Shanghai campus of the University of Science and Technology of China, where Alice and Bob aim to establish secret keys.

The polarization-entangled photon pairs in maximally entangled states in Alice’s and Bob’s sites are generated by pumping the periodically-poled MgO-doped lithium niobate (Type 0-PPMgLN) crystals in the dual-frequency Sagnac rings with two intrinsic phase-independent pulsed lasers at a 250 MHz repetition rate. We single out the signal (1556 nm)/idler (1560 nm) photons with cascaded dense wavelength-division multiplexing (DWDM) filters. To ensure high single-photon state purity, we further filter the photons by fiber Bragg gratings (FBGs) with a bandwidth of 3.3 GHz before the measurements. In both sites, we generate entangled photon pairs in state

(3) $$\begin{array}{c} |\phi^{+}\rangle \;=\frac{1}{\sqrt{2}}(|H\rangle |H\rangle +\;\left|V\rangle |V\rangle \right) \end{array}$$

where |H〉 and |V〉 denote the horizontal and vertical polarization states, respectively.

For ES-QKD, signal photons are sent to an untrusted relay, Eve, who performs BSM by using a sandwich structure composed of 50:50 beam splitters (BSs)—polarization beam splitters (PBSs)—50:50 BSs, which can discriminate the Bell states $|\Psi^{-}\rangle$ and $|\Psi^{+}\rangle$, and probabilistically eliminate the effect of multi-photons. Alice and Bob hold idler photons to randomly perform single-photon polarization projection measurements, respectively. Alice passively chooses the measurement basis $A_{i}$ by using a 1 × 3 fiber coupler, with each output port connected to a measurement basis determined by the combination of a polarization controller and a PBS. Similarly, Bob passively chooses the measurement basis $B_{i}$. The four-fold coincidence counts between the basis $A_{1}$, $B_{1}$ and BSM results are used to generate secret key, and the four-fold coincidence counts between the basis $A_{2}$, $A_{3}$, $B_{1}$, $B_{2}$ and BSM results are used to calculate CHSH value.

**Figure 3:**
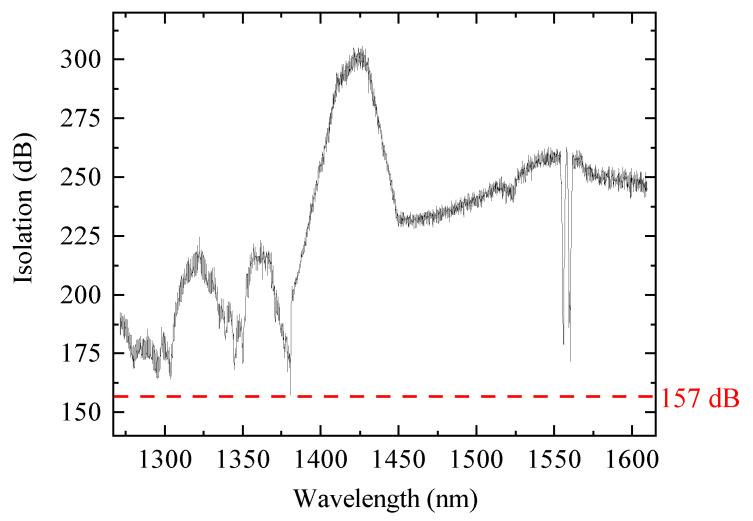
The isolation from the public quantum channel to the quantum channel of idler photons in the secure lab.

The experimental realization of the protocol presents several technical challenges. Firstly, to prevent Eve from attacking the ES-QKD system via pump pulses, we employ two independent gain-switched DFBs to generate entangled pairs in secure labs. Hence, we measure a CHSH game value after entanglement swapping, which puts stringent technological requirements on the interference between two independent sources and the high-quality entangled source. To realize the BSM, we synchronize the two independent sources, and reduce the distinguishability of the photons from Alice and Bob, especially the mode of spatial, polarization, spectrum, and temporal [35]. To obtain the high-brightness, high-efficiency and high-fidelity entangled source, we optimize the beam waists of the pump (779 nm) and down-conversion photons at the center of the Type 0-PPMgLN crystal [36], and finally we get the visibility of 0.97 at the average photon number per pulse of 0.02. Secondly, the entanglement sources isolate the quantum channel in the lab (Alice and Bob) from the public quantum channel in theory. Nevertheless, the isolation is limited by the imperfections of DWDMs. We increase the isolation to at least 157 dB in a wide spectrum, shown as Figure 3 (see Supplemental Materials). Thirdly, the secret key rate in the finite-data regime [37,38,39] is related to the sifted key rate and the error rate; hence, we optimize the average photon number per pulse and the ratio of each output port of the BSs at Alice’s and Bob’s parts. In addition, to produce a high rate and low error for detection, we employ 18 high-quality superconducting nanowire single-photon detectors (SNSPDs) with around 75% efficiency and a 100 Hz dark count rate on average.

**Figure 4:**
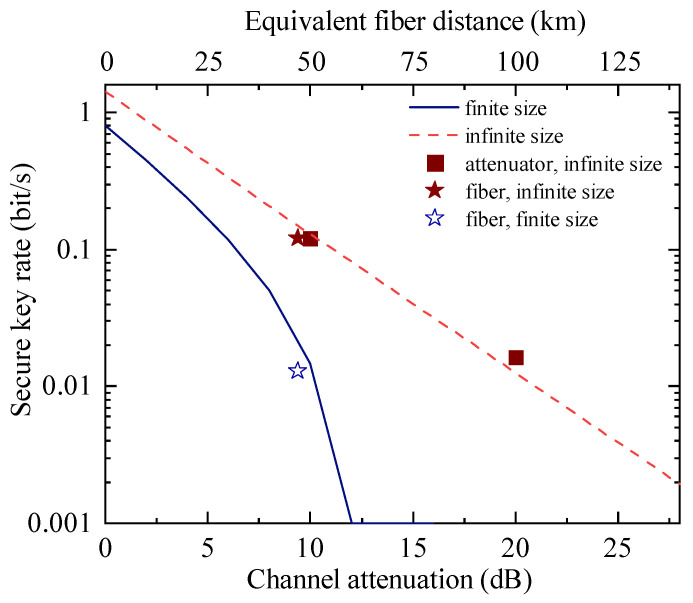
ESQKD secure key rates. The blue solid line and red dashed line are the simulation of the key rate with and without the finite-size analysis, respectively. The rates are plotted against the total attenuation (lower x-axis) and the equivalent fiber distance (upper x-axis) of the public quantum channel. Squares refer to key rates using the attenuators without the finite-size analysis. The solid and hollow stars are the key rate with and without the finite-size analysis obtained using two 25 km coiled fibers and the total attenuation is 9.4 dB.

For the ES-QKD demonstration, we run the system by using both variable attenuators and standard single-mode fiber. The experimental results for the attenuation of 10 and 20 dB (equivalent to 50 km and 100 km fibers) are shown in Figure 4. At 100 km (20 dB simulated loss), we collected 6304 bits of raw key over a 143,873 s acquisition time, obtaining a Bell violation value of S=2.575±0.095 and QBER of Q=0.0333±0.0033 for the BSM result of $|\Psi^{-}\rangle$, and S=2.659±0.092 and Q=0.0432±0.0038 for the BSM result of $|\Psi^{+}\rangle$. In the asymptotic case, the secret key rate is 0.0163 bps. Next, to replicate a realistic deployment scenario, we replaced the variable attenuators with two 25 km single-mode coiled fibers, resulting in a 50 km total fiber length and an attenuation of 9.4 dB. Over an acquisition time of 697,708 s, a total of 467,899 raw key bits were accumulated. The real optical fiber causes more uncertainty, such as the stabilities of polarization and time jitter of the channels, thus a slightly higher QBER. Even so, we still obtain the secret key rate of 0.121 and 0.0128 bits/s in the asymptotic and finite-key regimes, respectively. As theoretically expected, the secure key rate in the finite-size regime is noticeably lower than its asymptotic counterpart. This reduction occurs because the rigorous finite-key analysis must account for statistical fluctuations bounded by specific confidence intervals ($\delta_{est}$ and $\delta_{con}$) during the Bell test, as well as failure probabilities ($\epsilon_{EC}$ and $\epsilon_{PA}$) associated with post-processing. As detailed in Appendix A, these necessary penalty factors strictly reduce the final extractable secure key length to guarantee practical security, whereas they are intrinsically absent in the theoretical infinite-size limit.

## 4. Conclusions

To summarize, we have demonstrated ES-QKD based on the Bell test with two independent entangled photon sources in the field, up to 50 km of fiber, and 20 dB of attenuation. Entanglement swapping enables each user’s quantum state preparation and detection in a completely private space, where any probe from outside is isolated. This intrinsically protects against source-side attacks, offering a distinct advantage over measurement-device-independent QKD. Furthermore, by increasing the local detection efficiency, our experiment can be directly extended to a fully DI-QKD scenario with a local Bell test [40]. Thus, the present scheme is considerably less demanding experimentally than fully DI-QKD, occupying a highly practical intermediate regime for secure quantum networks.

Despite these advantages, the scalability of the current setup remains limited by technical imperfections, particularly the stringent spectral filtering required for high-visibility interference. Future improvements will require advanced source technologies, such as frequency-uncorrelated SPDC sources. Additionally, since the SPDC sources used in the present scheme are inherently broadband, these improvements can be seamlessly integrated with DWDM to execute parallel swapping processes, drastically multiplying the aggregate key rate. Looking further ahead, the intrinsic probabilistic limit of linear-optical Bell-state measurements can be circumvented by quantum-Zeno-based entanglement swapping, which approaches deterministic operation without controlled-NOT gates and is therefore an attractive building block for future quantum repeaters [41]. Together with quantum memories [42], these multiplexed and deterministic architectures can implement practical quantum repeaters and the quantum internet. Overall, our experiment marks a critical step towards the realization of a secure quantum network in the real world. 

## Data Availability

All data is available from the corresponding authors upon reasonable request.

## Supplementary Materials

The following supporting information can be downloaded at https://www.mdpi.com/article/10.3390/e28050518/s1, Figure S1: The experimental results of the visibility; Figure S2: The experimental result of HOM interference; Figure S3: Schematic of the simulated injection attack by Eve; Figure S4: Isolation of each device; Table S1: Details of the experimental results for 0 km; Table S2: Details of the experimental results for 50 km using a variable attenuator; Table S3: Details of the experimental results for 50 km using two 25 km coiled fibers; Table S4: Details of the experimental results for 100 km using a variable attenuator.

## Appendix A. Finite-Key Rate

For collective attacks [23], the finite key rates can be derived using the asymptotic equipartition property [43]. The protocol will generate a $\left(2\epsilon_{EC}+\epsilon_{s}+\epsilon_{PA}\right)$-correct-and-secret key of length [44],

(A1) $$\begin{array}{cc} l\geq & n[1-h\left(\frac{1}{2}+\frac{1}{2}\sqrt{\frac{1}{4}{\left(S-\delta_{est}-\delta_{con}\right)}^{2}-1}\right)-h\left(Q\right)]\\ \; & -\sqrt{n}\left(4\log_{2}\left(2\sqrt{2}+1\right)\left(\sqrt{\log_{2}\frac{2}{\epsilon_{s}^{2}}}+\sqrt{\log_{2}\frac{8}{{\epsilon^{\prime }}_{EC}^{2}}}\right)\right)\\ \; & -\log_{2}\left(\frac{8}{{\epsilon^{\prime }}_{EC}^{2}}+\frac{2}{2-\epsilon_{EC}^{\prime }}\right)-\log_{2}\frac{1}{\epsilon_{EC}}-2\log_{2}\frac{1}{2\epsilon_{PA}}, \end{array}$$

or it aborts with a probability higher than $1-\epsilon_{con}-\epsilon_{EC}$. Here, *n* is the accepted number of key generation rounds, and *h* is the binary entropy, $h\left(x\right)=-x\log_{2}x-\left(1-x\right)\log_{2}\left(1-x\right)$. The parameter $\epsilon_{EC}$ is the failure probability of error correction conditioned on not aborting, namely the probability that Alice and Bob still hold different keys after error correction. The parameter $\epsilon_{EC}^{\prime }$ is an auxiliary smoothing parameter entering the finite-size upper bound on the information leakage during error correction. The quantity $\epsilon_{s}$ is the smoothing parameter in the smooth min-entropy, which controls the allowed deviation to a nearby typical state in the secrecy analysis. Finally, $\epsilon_{PA}$ is the failure probability of privacy amplification, i.e., the probability that the extracted key is not perfectly uniform and secret [44].

The statistical error $\delta_{est}=\sqrt{\frac{\ln(1/\epsilon_{est})}{2m}}$ is the statistical deviation of the Bell test for an honest implementation with m test rounds, whereas $\delta_{con}=\sqrt{\frac{\ln(1/\epsilon_{con})}{2m}}$ is the confidence interval used to infer a lower bound on the underlying Bell parameter under collective attacks from the observed test statistics.

In the numerical evaluation, we set the confidence parameters for parameter estimation and Bell estimation to $\epsilon_{est}=\epsilon_{con}=5\times 10^{-4}$, and fix the remaining correctness and secrecy budget according to $2\epsilon_{EC}+\epsilon_{s}+\epsilon_{PA}=2\times 10^{-6}$.

## Appendix B. Modelling ES-QKD Scheme

In the ES-QKD protocol, Alice and Bob each possess an entangled source, denoted by $S_{A}$ and $S_{B}$, respectively. The state generated by Alice’s source $S_{A}$ is written as

(A2) $$|\psi_{A}\rangle \;=\sqrt{\left(1-\tanh^{2}\left(g\right)\right)\left(1-\tanh^{2}\left(\bar{g}\right)\right)}\;\exp\;\left(\tanh\left(g\right)\;a_{l}^{†}c_{a⊥}^{†}-\tanh\left(\bar{g}\right)\;a_{l⊥}^{†}c_{a}^{†}\right)|0\rangle .$$

Here, $\left\{a_{l},a_{l⊥}\right\}$ denote two orthogonal bosonic modes retained by Alice, while $\left\{c_{a},c_{a⊥}\right\}$ are the modes sent to an untrusted central node, where a Bell-state measurement is performed. In the worst-case scenario, this node is controlled by Eve. Bob is described analogously: he keeps the modes $\left\{b_{l},b_{l⊥}\right\}$ for local polarization measurements and sends the modes $\left\{c_{b},c_{b⊥}\right\}$ to the central node. The parameters *g* and $\bar{g}$ characterize the two squeezing amplitudes associated with the two orthogonal polarization-mode pairs. In particular, *g* corresponds to the pair $a_{l}^{†}c_{a⊥}^{†}$, whereas $\bar{g}$ corresponds to $a_{l⊥}^{†}c_{a}^{†}$. For a given two-mode squeezed contribution with squeezing parameter *g*, the mean photon number is $\langle \psi |a^{†}a|\psi \rangle =\sinh^{2}\left(g\right)$.

In experiments, the photon detectors do not resolve the photon number. The heralding efficiency from photon creation to detection is denoted as η, and the dark count probability is pd. The operator for a click is given by $D\left(O\right)=1-D_{nc}\left(O\right)$, where $D_{nc}\left(O\right)=\left(1-pd\right){\left(1-\eta \right)}^{O^{+}O}$, and *O* is the corresponding bosonic operators. In experiments, Alice and Bob will choose randomly and independently from the measurement settings $A_{i}\in \left\{\sigma_{z},\frac{1}{\sqrt{2}}\left(\sigma_{z}-\sigma_{x}\right),\frac{1}{\sqrt{2}}\left(\sigma_{z}+\sigma_{x}\right)\right\}$ and $B_{j}\in \left\{\sigma_{z},\sigma_{x}\right\}$, respectively, where $\sigma_{z}$ and $\sigma_{x}$ are two Pauli matrices. After their measurements, the sates that are sent to the untrusted node can be written as

(A3) $$\begin{array}{cc} \; & |\psi_{C}^{a}\rangle \;=\mathrm{Tr}[D\left(A_{a|i}\right)D_{nc}\left(A_{\bar{a}|i}\right)|\psi_{A}\rangle \langle \psi_{A}|],\\ \; & |\psi_{C}^{b}\rangle \;=\mathrm{Tr}[D\left(B_{b|i}\right)D_{nc}\left(B_{\bar{b}|i}\right)|\psi_{B}\rangle \langle \psi_{B}|], \end{array}$$

where, $A_{a|i}$ ($B_{b|i}$) is the projection operator of obtaining the outcome *a* (*b*) from the measurement $A_{i}$ ($B_{i}$), and $D\left(O\right)D_{nc}\left(O_{⊥}\right)$ means that only one detector clicks on each side. If the BSM succeed at the ESR node, Alice and Bob accept the round.

Since the probability that Alice and Bob both choose the key generation measurement is (1−λ)(1−λ/2), there will be N(1−λ)(1−λ/2) number of rounds for key generation. Here, *N* is the total number of rounds in the experiment. When the channel attenuation is 10 dB and $N=1.44\times 10^{14}$, the numerical result shows λ=0.3, while the mean number of photons of the source should be set to 0.028.
