# Supplementary material for: Entanglement Swapping Enables the Practical Security of Quantum Cryptography
Source: Entropy (Basel). 2026 May 4;28(5):518. doi: 10.3390/e28050518 (PMC13205301; doi:10.3390/e28050518)
Supplement: Supplementary file 1 [file entropy-28-00518-s001.zip › entropy-4258670-supplementary.pdf]

Supplementary Material for Entanglement swapping enables the practical security of quantum cryptography

This PDF file includes details of the experiment:

- **Independent polarization entangled photon-pair sources**
- **The isolation of the quantum channel in the lab (Alice and Bob) from the public quantum channel**
- **The setup of Bell state measurement**
- **Details of the experimental results**

1 Independent polarization entangled photon-pair sources

In this experiment, we prepare two independent polarization entangled photon-pair sources in Bell state $|\Phi^+\rangle = \frac{1}{\sqrt{2}}(|H\rangle|H\rangle + |V\rangle|V\rangle)$ with the setup shown in FIG.2(b) of the main text, where $|H\rangle$ and $|V\rangle$ denote horizontal and vertical polarization states, respectively. Since the generated

photon pairs are polarization degenerate, the signal and idler photons are separated by two cascaded dense wavelength division multiplexing filters (DWDMs). Using the optimal method in Ref [1], we set the pump beam waist and the collection beam waist to 55 μm and 54 μm respectively, to achieve a balance between the pair generation probability and heralding efficiency. Furthermore, we pass the photon pairs through the 3.3 GHz fibre Bragg gratings (FBGs) to suppress the spectral distinguishability. And the single-mode fiber eliminates the spatial distinguishability. The overall detection efficiency for a single photon from the creation to detection (including heralding ratio, channel loss, filtering loss and measurement loss) are measured to be 12% and 10%, at Alice's (or Bob's) and Eve's detector, respectively.

To characterize the generated entangled state, we measure the visibility in $|H\rangle$ /$|V\rangle$ basis and $|+\rangle$/$|-\rangle$ basis. As shown in Fig. S1, we obtain the average visibility of (97.29±0.07)% at Alice's Lab and (97.70±0.07)% at Bob's Lab with an average number of pairs per pulse of 0.02.

To characterize the interference of the two independent sources, we measure the visibility of Hong-Ou-Mandel interference. To suppress the temporal distinguishability, we synchronize the two sources. The pulse pattern generator (PPG) at Alice's Lab is the master clock, which generates a 12.5 GHz sinusoidal signal to synchronize the source at Bob's Lab. The root-mean-square (RMS) value of the time jitter between the two pump lasers is about 4 ps, which is much smaller than the 133 ps coherent time of the signal photons. After suppressing the distinguishability of photons in spectral, spatial, temporal, and polarization modes, we obtain a fitted visibility of 93.2$\pm$0.3%, shown in Fig. S2.

2 The isolation of the quantum channel in the lab (Alice and Bob) from the public quantum channel

The entangled photon-pair sources isolate the quantum channel in the Lab (Alice and Bob) from the public quantum channel naturally. Here, the quantum channel denotes the channel that transfers entangled photons. Nevertheless, the isolation is limited by the imperfection of DWDMs. Therefore, we add a wavelength division multiplexing filter (WDM) and a circulator (CIR) to each side, shown in Figure 2(b) of the main text. We simulate the process in which Eve attacks the measurement in the lab from the public quantum channel, as shown in Fig. S3. The light source, which is generated by Eve, must travel through the following devices: a CIR, a WDM, two cascaded DWDMs and an FBG. We test the isolation of each device by comparing the power of a broadband laser before and after the device with a spectroscope. For overall isolation, we add the isolation of

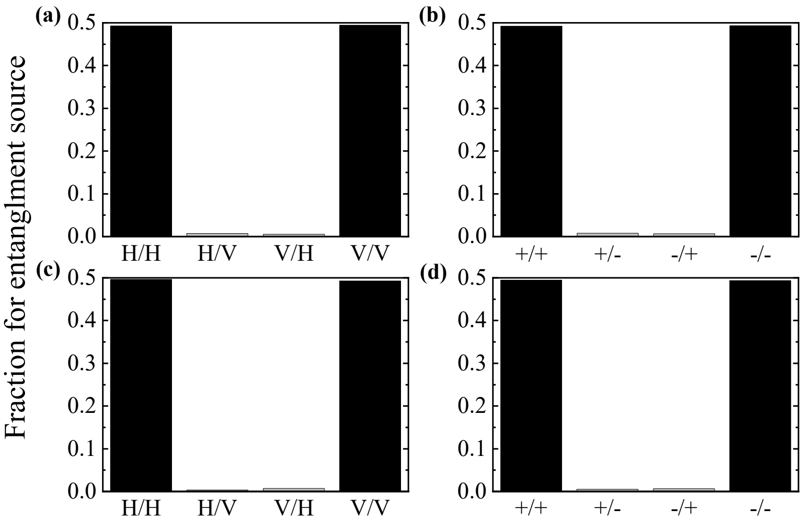

Figure S1: **The experimental results of the visibility.** (a) and (b) show the experimentally measured fractions both in the $|H\rangle$ /$|V\rangle$ basis and the $|+\rangle$/$|-\rangle$ basis for the states of the sources at Alice's Lab, and (c) and (d) are for Bob. The average visibility are (97.29±0.065)% and (97.70±0.07)%, respectively.

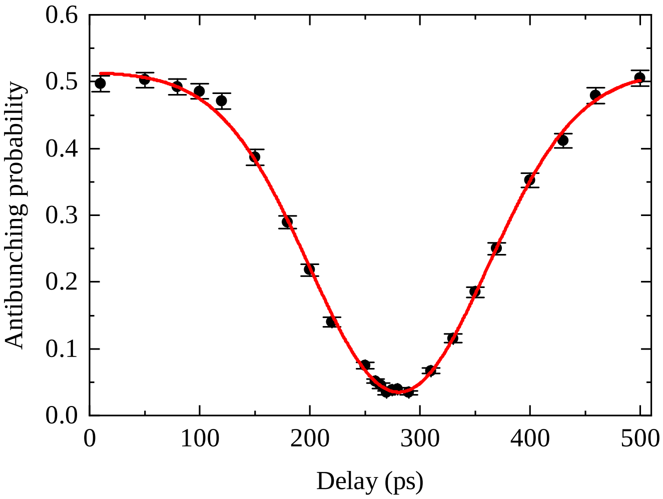

Figure S2: **The experimental result of HOM interference.** Each data point is accumulated for more than 1200 photon counts. The visibility of the fitted curve is $(93.2 \pm 0.3)\%$.

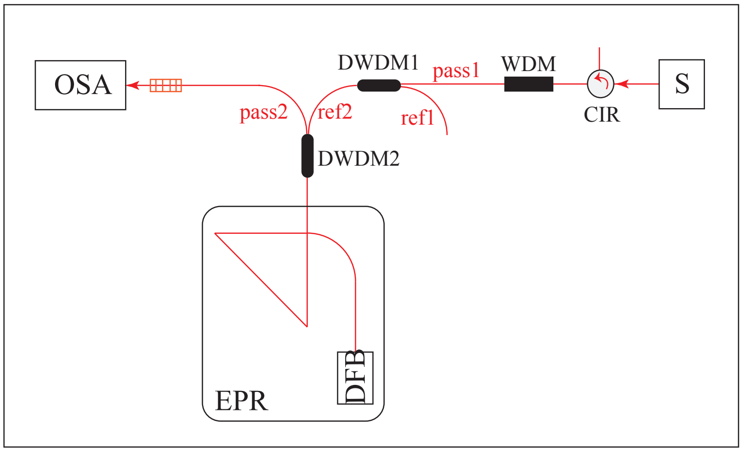

Figure S3: **Schematic of the simulated injection attack by Eve.** Eve uses a light source (S) to inject into the quantum channel. We use an optical spectrum analyzer (OSA) to record the wavelength and power. CIR: circulator; WDM: wavelength division multiplexing filter; DWDM: dense wavelength division multiplexing filter; EPR: the EPR source in our experiment.

all the devices up. Fig. S4 shows the isolation for each device and Figure 3 of the main text shows the overall result.

3 The setup of Bell state measurement

As depicted in Figure 2 of the main text, the Bell state measurement (BSM) at Eve's node is realized using a network of beam splitters (BSs) and polarizing beam splitters (PBSs). Photons arriving from the two sources undergo interference at the primary BS. Specifically, photon pairs in the anti-symmetric Bell state $|\Psi^-\rangle$ emerge from distinct output ports of the BS. In contrast, pairs in the symmetric $|\Psi^+\rangle$ state exhibit bunching and exit from the same BS port, but are deterministically separated into different ports by the subsequent PBSs. Finally, additional BSs are utilized before the detectors to suppress the impact of multi-photon emissions.

4 Details of the experimental results

The experimental performance is evaluated using variable attenuators to simulate the transmission losses of standard single-mode fibers (0.2 dB/km) at 0 km, 50 km, and 100 km. To replicate a realistic deployment scenario, the system is further tested using two 25 km single-mode coiled fibers, forming a 50 km total fiber channel. Detailed experimental results are summarized in Tables S1-S4.

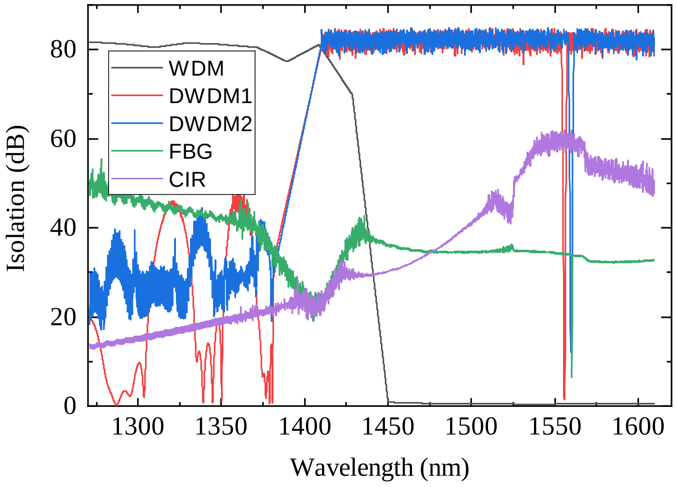

Figure S4: **Isolation of each device.**

In terms of data processing, coincident detection counts obtained when Alice and Bob both measure in the σ_z basis are defined as the key generation rounds, representing the raw key length (bits). Coincident counts obtained in bases required for Bell tests, $\sigma_z \pm \sigma_x$ for Alice and σ_z or σ_x for Bob, are defined as the Bell test rounds, which are utilized to calculate the CHSH violation value and bound the eavesdropper's information.

The primary experimental parameters are as follows: the mean photon number per pulse is $\mu = 0.028$, and the system repetition rate is $f_{rep} = 250$ MHz. The single-arm heralding efficiencies for the entangled sources at Alice and Bob are estimated as $\eta_A \approx \eta_{E1} \approx 0.12$ and $\eta_B \approx \eta_{E2} \approx 0.10$, respectively. Taking the 10 dB channel attenuation (equivalent to 50 km) as a representative case with a total transmittance $T_{channel} = 0.1$, the theoretical four-fold coincidence rate (R_{4fold}) is given by:

$$R_{4fold} = \mu^2 \cdot (\eta_A \cdot \eta_{E1}) \cdot (\eta_B \cdot \eta_{E2}) \cdot T_{channel} \cdot f_{rep} \approx 2.82 \text{ counts/s.} \tag{S1}$$

By factoring in the Bell state measurement success probability ($P_{BSM} = 1/2$) and the basis reconciliation probability (γ), the theoretical raw key generation rate (R_{raw}) is determined. In our setup, Alice uses a beam splitter with a splitting ratio of 1.5:1.5:7 to passively choose her measurement basis (assigning 15% to $\sigma_z + \sigma_x$, 15% to $\sigma_z - \sigma_x$, and 70% to σ_z), while Bob uses a 15:85 beam splitter (assigning 15% to σ_x and 85% to σ_z). Therefore, the probability that both parties choose the σ_z basis for key generation is $\gamma = 0.70 \times 0.85 = 0.595$. The theoretical raw key

generation rate is:

$$R_{raw} = R_{4fold} \cdot P_{BSM} \cdot \gamma \approx 0.84 \text{ bits/s}. \tag{S2}$$

Experimentally, the average raw key generation rate measured at 50 km attenuation is approximately 0.575 bits/s (as shown in Table S2). This experimental value shows reasonable agreement with the theoretical estimation (~ 0.84 bits/s). The minor discrepancy is primarily attributed to additional insertion losses introduced by practical optical connections and slight fluctuations of the actual mean photon number from its nominal value.

1. Bennink, R.S. Optimal collinear Gaussian beams for spontaneous parametric down-conversion. Physical Review A **2010**, 81, 053805. https://doi.org/10.1103/physreva.81.053805.

Table S1: Details of the experimental results for 0 km. Acquisition time is 30882 s. The error bars represent one standard deviation.

BSM result	$\|\Psi^-\rangle$	$\|\Psi^+\rangle$
Bell test rounds	53069	53150
CHSH value	2.593 ± 0.018	2.552 ± 0.018
Key generation rounds (raw key length)	84910	86010
Quantum bit error rate (Q)	0.0385 ± 0.0007	0.0387 ± 0.0007
Key rates with (without) the finite-size analysis	0.25 (1.7) bit/s	

Table S2: Details of the experimental results for 50 km using a variable attenuator. Acquisition time is 579737 s. The error bars represent one standard deviation.

BSM result	$\|\Psi^-\rangle$	$\|\Psi^+\rangle$
Bell test rounds	102719	102993
CHSH value	2.518 ± 0.013	2.448 ± 0.014
Key generation rounds (raw key length)	166814	166613
Quantum bit error rate (Q)	0.0413 ± 0.0005	0.0426 ± 0.0005
Key rates with (without) the finite-size analysis	0.01 (0.12) bit/s	

Table S3: Details of the experimental results for 50 km using two 25 km coiled fibers. Acquisition time is 697708 s. The error bars represent one standard deviation.

BSM result	$\|\Psi^-\rangle$	$\|\Psi^+\rangle$
Bell test rounds	142248	143077
CHSH value	2.485 ± 0.015	2.485 ± 0.015
Key generation rounds (raw key length)	233799	234100
Quantum bit error rate (Q)	0.0427 ± 0.0004	0.0445 ± 0.0004
Key rates with (without) the finite-size analysis	0.0128 (0.121) bit/s	

Table S4: Details of the experimental results for 100 km using a variable attenuator. Acquisition time is 143873 s. The error bars represent one standard deviation. Note: Due to the limited raw key size at this distance, the finite-size key rate drops to zero. Therefore, only the asymptotic key rate is presented.

BSM result	$\|\Psi^-\rangle$	$\|\Psi^+\rangle$
Bell test rounds	1901	1877
CHSH value	2.575 ± 0.095	2.659 ± 0.092
Key generation rounds (raw key length)	3178	3126
Quantum bit error rate (Q)	0.0333 ± 0.0033	0.0432 ± 0.0038
Key rates with the infinite-size analysis	0.0163 bit/s	
